# Pseudo-Meigs’ Syndrome With Eosinophilic Pleural Effusion

**DOI:** 10.7759/cureus.54686

**Published:** 2024-02-22

**Authors:** Masafumi Shimoda, Yoshiaki Tanaka, Kozo Morimoto, Iori Moue, Ken Ohta

**Affiliations:** 1 Respiratory Disease Center, Fukujuji Hospital, Kiyose, JPN

**Keywords:** pleural effusion, eosinophilia, teratoma, eosinophilic pleural effusion, pseudo-meigs’ syndrome

## Abstract

We present a rare case of a 45-year-old woman with pseudo-Meigs' syndrome and eosinophilic pleural effusion (EPE). She experienced cough, sputum, and dyspnea with a large right pleural effusion. Laboratory tests showed eosinophilia in the blood and pleural fluid. An ovarian tumor and ascites were also detected. After left salpingo-oophorectomy, the tumor was diagnosed as a mature cystic teratoma of the left ovary. The right-sided pleural effusion gradually resolved. Pseudo-Meigs' syndrome is characterized by benign ovarian tumor, ascites, and pleural effusion. Typically, it is associated with exudate pleural effusion characterized by a predominance of mononuclear cells. The occurrence of eosinophilic pleural effusion in our patient may be exceptionally rare.

## Introduction

Pseudo-Meigs’ syndrome is a rare disease characterized by the presence of pleural effusion and ascites caused by pelvic or abdominal tumors [1,2]. Unlike Meigs' syndrome, which is associated with benign fibroma or fibroma-like ovarian tumors (such as thecoma, granulosa cell tumor, or Brenner tumor), pseudo-Meigs' syndrome is caused by other types of benign or malignant tumors in the pelvic or abdominal region [1]. While pseudo-Meigs' syndrome is well-defined, certain aspects, particularly related to pleural effusion, remain unclear [1]. Pleural effusion in this syndrome is typically exudative and benign, resolving after tumor removal with a favorable prognosis [1,2]. However, the laboratory findings and cell fractionation of this pleural effusion have not been extensively studied. Here, we report a rare case of pseudo-Meigs' syndrome with eosinophilic pleural effusion (EPE).

## Case presentation

A 45-year-old woman, who was a cashier, presented with a three-week history of cough, sputum, and dyspnea. She had no significant medical or smoking history. For the past four years, she had been taking over-the-counter ibuprofen for headaches, typically once every two to three days; however, she had not been on any regular medication. She had a history of allergies, including hay fever. After visiting a local doctor, chest radiography revealed the presence of right pleural effusion, prompting her to seek further evaluation and treatment at our hospital. Her vital signs were within normal limits, with no signs of respiratory failure, and physical examination revealed no abnormalities, except for decreased breath sounds in the right lower lung. Laboratory results indicated eosinophilia, elevated C-reactive protein levels, and increased carbohydrate antigen 125 (CA125) levels (Table 1).

**Table 1:** Transition of laboratory findings in the patient * No established normal values for the pleural effusion test WBC: White blood cells; CRP: C-reactive protein; CA125: Carbohydrate antigen 125; TP: Total protein; LDH: Lactate dehydrogenase; ADA: Adenosine deaminase

	First visit	Three weeks later	Four weeks later	Four months later (post-operation)
Peripheral blood and serology (unit) (normal range)				
WBC (/µL) (3500-9100)	9560	5360	5850	3560
Neutrophils (/µL) (1720-6390)	6214	3982	4265	2560
Lymphocytes (/µL) (1200-3690)	860	783	819	801
Eosinophils (/µL) (70-440)	2247	332	538	61
CRP (mg/dL) (≤0.14)	7.76	4.51	0.64	0.03
CA125 (U/mL) (≤35.0)	174			14.5
Pleural effusion (unit)*				
Cell fractionation				
Neutrophils (%)	20.5	-	-	-
Lymphocytes (%)	27.0	87.0	95.5	-
Eosinophils (%)	40.5	6.0	2.0	-
Methothelial cell (%)	few	0.5	few	
TP (g/dL)	5.00	4.86	4.66	-
LDH (IU/L)	886	346	358	-
ADA (U/L)	37	30	27	-

**Figure 1:**
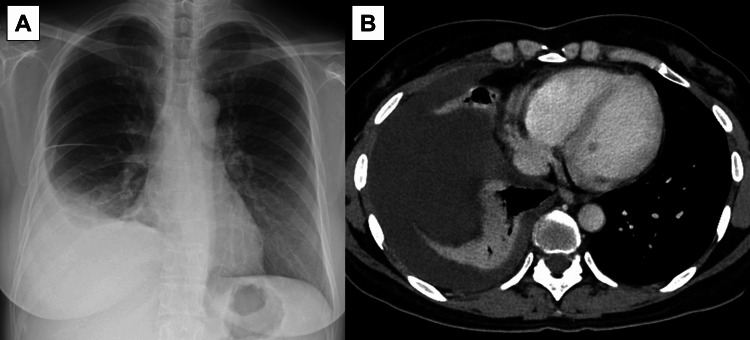
Chest radiography (A) and computed tomography scans (B) showed a large amount of right pleural effusion without any lung lesions

She had negative antinuclear antibodies, anti-cyclic citrullinated peptides antibodies, antineutrophil cytoplasmic antibodies, anti-Sjögren’s-syndrome-related antigen A, and immunoglobulin (Ig) G4. The serum allergen-specific IgE test indicated a positive result for cedar pollen (class 3), while other allergens had negative results. Serum parasite antibodies were negative results. Chest radiography and computed tomography (CT) scans revealed a large right pleural effusion without any lung lesions including pulmonary embolism (Figure 1).

**Figure 2:**
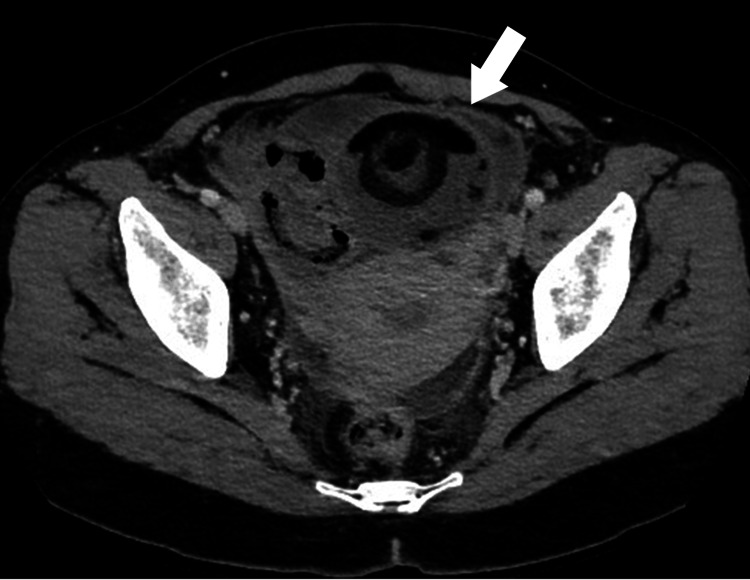
Abdominal-pelvic contrast-enhanced computed tomography showed a left ovarian tumor and ascites in the pelvis (arrow)

During the initial chest CT at our hospital, coincidental enhancement of intra-abdominal soft tissue shadow was noted, prompting an abdominal-pelvic contrast-enhanced CT. This revealed a left ovarian tumor and ascites in the pelvis (Figure 2).

The pleural effusion obtained through thoracentesis was characterized as exudative, with an increased eosinophil ratio. Bacterial culture, tuberculosis examinations (smear and polymerase chain reaction tests), and cytology of the pleural fluid revealed an accumulation of eosinophils with reactive mesothelial cells, with no presence of malignant cells. Her serum eosinophils decreased in parallel with pleural eosinophils, although her serum eosinophils remained elevated at 538/µL. Despite receiving no treatment, the white blood cell count and C-reactive protein gradually decreased after thoracentesis. However, the pleural effusion recurred, and repeated thoracentesis for diagnostic and therapeutic purposes showed a shift towards lymphocyte predominance, although the pleural effusion remained exudative (Table 1).

She was subsequently referred to another gynecology hospital, where a left salpingo-oophorectomy was performed five weeks after her first visit to our hospital. Pathological examination of the tumor revealed a mature cystic teratoma of the left ovary. Gradual resolution of the right-sided pleural effusion was observed. Although double-stranded deoxyribonucleic acid was not examined, symptoms and signs indicated of autoinflammatory or immune causes were absent, and there was no history of trauma, asbestos exposure, or other causes of EPE. Therefore, she was diagnosed with pseudo-Meigs’ syndrome. Serum eosinophils levels and CA125 decreased to within the normal range. Subsequently, she has been progressing without any recurrence, despite restarting non-steroidal anti-inflammatory drugs (NSAIDs) such as loxoprofen and ibuprofen after the operation, which had been discontinued since her first visit to our hospital.

We obtained informed consent from the patient for the publication of this case.

## Discussion

We present a rare case of pseudo-Meigs’ syndrome accompanied by concomitant EPE. Interestingly, the initial thoracentesis revealed a high concentration of eosinophils in the pleural fluid, while subsequent thoracentesis showed a shift to a lymphocytic predominance. To the best of our knowledge, pseudo-Meigs’ syndrome has not been previously reported to be associated with an elevation of pleural eosinophils, unlike our case. We have formulated three hypotheses for the EPE: the first involves an association with eosinophilia, the second suggests tumor-associated EPE and the third considers drug-induced EPE due to ibuprofen. However, there are limited reports on the characteristics of pleural effusion in pseudo-Meigs’ syndrome, leaving the exact cause of eosinophilic pleural effusion uncertain.

The etiology of EPE has been reported to include various causes such as blood/air in pleural space, infection, uremic pleuritis, malignancy, autoimmune disorders, drug reactions, acute/chronic eosinophilic pneumonia, hypereosinophilic syndrome, idiopathic cases, and others [3]. Blood eosinophilia can be found in many of those cases, particularly in more than half of idiopathic EPE [3]. However, to the best of our knowledge, blood eosinophilia is relatively rare in patients with pseudo-Meigs’ syndrome, with only one case showing increasing eosinophil count (7.2% {402/µL} eosinophils) in the context of pseudo-Meigs’ syndrome [4]. In our patient, although she had a history of hay fever, her serum allergen-specific IgE was mildly elevated for cedar pollen only, and she did not show obvious allergic symptoms. Moreover, her serum and pleural eosinophils decreased after the initial thoracentesis. It is hypothesized that pseudo-Meigs’ syndrome might be combined with EPE due to ibuprofen. It is important to note that she took ibuprofen intermittently and had maintained the same frequency for the past four years. Although our patient undergoes a health check-up once a year, no abnormalities, including eosinophilia, have ever been noted in the past. Furthermore, the patient has been progressing without any recurrence despite restarting NSAIDs. Therefore, we believed that the cause of EPE in our patient was not associated with drug-induced factors. Additionally, previous reports have shown that pleural eosinophil levels can decrease after repeated thoracentesis. In 38.5-66.7% of patients with EPE, EPE is detected only in the first pleural fluid samples [5,6]. However, the cause of EPE in patients with decreasing pleural eosinophil levels after repeated thoracentesis is not described in those reports. Therefore, in our patient, the change in pleural eosinophils may not be special and could occur regardless of the cause.

Another hypothesis is tumor-associated EPE. Nonmyeloid malignancies can lead to secondary eosinophilia through the production of cytokines, such as interleukin-3, interleukin-5, and granulocyte-macrophage colony-stimulating factor, which promote eosinophil differentiation and survival [7]. Many types of malignancies have been associated with tumor-associated EPE, however, pseudo-Meigs' syndrome is not included among these [3]. Regarding benign tumor-associated EPE, a previous report demonstrates a 17-year-old female with a benign cystic teratoma of the anterior mediastinum who presented with EPE [8]. The histological characteristics of the tumor in this case are similar to those of our patient. However, there are no reports of EPE in the context of pseudo-Meigs’ syndrome. In the context of Meigs’ syndrome, cases of EPE have been reported, with an estimated incidence of Meigs’ syndrome at approximately 1.9-2.0% among EPE patients [9,10]. While data on the incidence of pseudo-Meigs' syndrome are lacking, it is generally considered rarer than true Meigs' syndrome [11]. Therefore, EPE in patients with pseudo-Meigs' syndrome might not have been reported due to its rarity. 

In the four case reports of pseudo-Meigs’ syndrome that provided detailed information on pleural effusion cell fractionation, a prevailing characteristic was the presence of exudate and a predominance of mononuclear cells, mainly lymphocytes and/or mesothelial cells [12-15]. Serum CA125 levels, a biomarker for ovarian cancer, are often elevated in pseudo-Meigs’ syndrome cases. It is believed that CA125 elevation is due to inflammation and secretion from mesothelium cells, although the exact reason remains unclear [16]. In our patient, pleural mesothelial cells were very few despite the elevation of serum CA125 levels. The connection between EPE, the low count of pleural mesothelial cells, and the elevation of CA125 levels in our patient was uncertain. However, there has been a documented case where CA125 levels were elevated without an increase in mesothelial cells [14]. Additionally, cytological analysis of the pleural fluid from our patient revealed reactive mesothelial cells. While the exact mechanisms are still not fully understood, the improvement following ovarian tumor surgery and the exclusion of other potential causes suggest the involvement of EPE and pseudo-Meigs’ syndrome. EPE can have various causes, but our findings indicate that pseudo-Meigs’ syndrome might be a contributing factor.

This case did not undergo a pleural biopsy. The definition of pseudo-Meigs’ syndrome requires negative pleural fluid cytology and/or no malignant involvement in biopsy samples [1]. In our patient, pleural fluid cytology yielded negative results, and her pleural effusion showed spontaneous resolution after the resection of the ovarian tumor, leading to the diagnosis of pseudo-Meigs’ syndrome. Furthermore, the reason for the decrease in peripheral eosinophilia after the first thoracentesis remains uncertain.

## Conclusions

We report a rare case of pseudo-Meigs’ syndrome with eosinophilic pleural effusion and provide insights into the characteristics of pleural effusion in cases of pseudo-Meigs’ syndrome. Typically, pseudo-Meigs’ syndrome is associated with exudate pleural effusion characterized by a predominance of mononuclear cells. The occurrence of eosinophilic pleural effusion in our patient may be exceptionally rare. 
